# Insights into uveitis from Brentuximab vedotin in refractory Hodgkin’s lymphoma: a case report and brief review

**DOI:** 10.3389/fonc.2024.1419145

**Published:** 2024-08-05

**Authors:** Mengyuan Liu, Kexing Ren, Ping Ai, Liqun Zou

**Affiliations:** ^1^ Department of Radiation Oncology, Department of Head and Neck Oncology, Cancer Center, West China Hospital, Sichuan University, Chengdu, China; ^2^ Department of Medical Oncology, Cancer Center, West China Hospital of Sichuan University, Chengdu, China

**Keywords:** Brentuximab vedotin, Hodgkin’s lymphoma, uveitis, MMAE, antibody-drug conjugate, case report

## Abstract

This case report describes a 16-year-old patient with refractory Hodgkin’s lymphoma who developed bilateral anterior and intermediate uveitis as an adverse reaction to Brentuximab vedotin (BV). This is a rare case of an ocular adverse reaction potentially related to BV, with symptoms like blurred vision, decreased visual acuity, photophobia, and redness. Potential mechanisms include BV targeting CD30+ T cells in the uveal tissue or an immune response triggered by the microtubule-disrupting agent MMAE within BV. This highlights the need for vigilant monitoring of ocular adverse events in BV-treated patients and further research into their mechanisms and management.

## Introduction

BV is an antibody-drug conjugate (ADC) that targets CD30, a protein expressed on the surface of cancer cells in Hodgkin’s lymphoma (HL) and systemic anaplastic large cell lymphoma (ALCL). It consists of a monoclonal antibody (cAC10), a microtubule-disrupting agent (MMAE) (1), and a protease-cleavable linker. Approved by the FDA in August 2011, BV treats HL and systemic ALCL (2), and since March 2018, it is also approved for use with chemotherapy in stage III or IV classical Hodgkin’s lymphoma (cHL) (3).

The most frequently observed adverse reactions (≥20%) associated with this drug include neutropenia, anemia, peripheral sensory neuropathy, nausea, malaise, constipation, diarrhea, vomiting, and fever. While the safety analysis of BV did not report uveitis and the FDA labeling does not specifically address ocular adverse events, only a few case reports have documented in the literature (4–8).

## Case report

**Figure 1:**
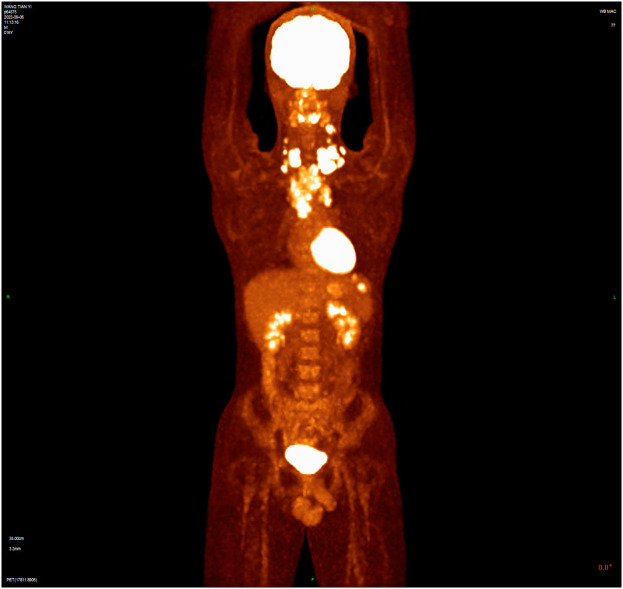
The baseline PET/CT shows that tumor had invaded the cervical and thoracic lymph nodes, spleen, and three cervical vertebrae.

**Figure 2:**
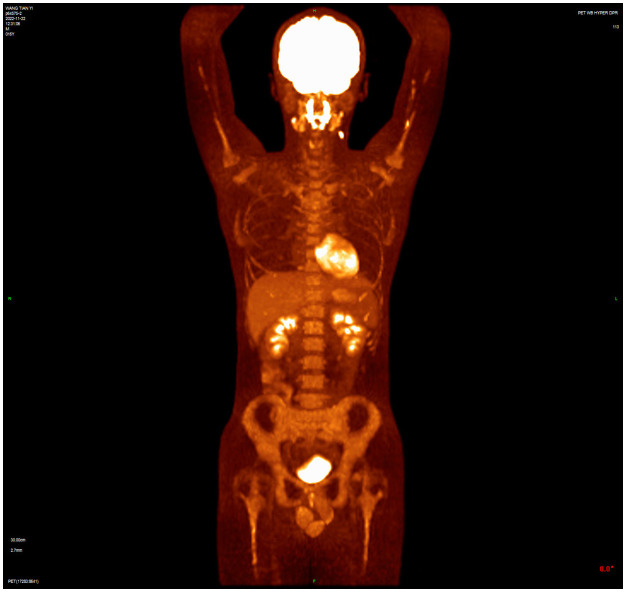
PET/CT after two cycles of treatment showed incomplete resolution of the lesion, with increased lymph node uptake of 18F-FDG in the left posterior cervical triangle.

A 16-year-old male presented with a painless neck mass in the left neck without B symptoms (fever, night sweats, or weight loss), in September 2022 at the outpatient clinic of West China Hospital, Sichuan University. PET/CT revealed multiple enlarged lymph nodes in the bilateral neck, posterior cervical triangle, supraclavicular fossa, and mediastinum (SUVmax 13.89). It also showed two hypodense lesions in the spleen (SUVmax 6.81) and bone density reduction in three cervical vertebrae (SUVmax 8.99) (**Figure 1**). Immunohistochemistry of the neck mass biopsy indicated classical Hodgkin’s lymphoma: LCA (–), CD20 (–), CD3 (–), CD30 (+), CD15 (+, individual), PAX-5 (+, weak), MUM1 (+), PD-1 (–), EBV (–), ALK (OTI1H7) (–), CD68PG-M1 (–). Bone marrow aspiration flow cytometry (FCM) showed no significant abnormalities. The disease was classified as Advanced Stage (AS) according to the GHSG staging system (Lugano stage IVS, group A, IPS score 2) (9–11). “Group A” refers to the absence of systemic symptoms such as fever, night sweats, and weight loss, which are collectively known as B symptoms (12). Due to the patient’s age and reproductive toxicity of bleomycin, bleomycin was excluded from treatment after consultation with the patient’s guardian. The patient was treated with Brentuximab vedotin, pirubicin, vincristine, and dacarbazine (A+AVD). One month after the first cycle of BV, the patient experienced photophobia and conjunctival redness. Visual acuity was 0.8 in the right eye and 0.6 in the left eye. Slit-lamp examination showed mixed hyperemia and corneal dust KP+ in both eyes. Bilateral uveitis was diagnosed and managed with Tobramycin Dexamethasone Eye Drops (Tobradex) and Diclofenac Sodium Eye Drops. Although the symptoms were initially relieved, the patient returned to the clinic the day after the second cycle of BV treatment with complaints of photophobia and redness in both eyes. Local treatment was administered, and the symptoms were alleviated. Complete response was not achieved after two cycles of treatment (**Figure 2**). Patients receiving BV+Nivo which are used in salvage therapy for cHL and then proceeding to Allogeneic Hematopoietic Cell Transplantation (AHCT) have a 91% 3-year PFS rate (13). Therapy was switched to the PD-1 inhibitor tislelizumab combined with BV for further salvage treatment.

**Figure 3:**
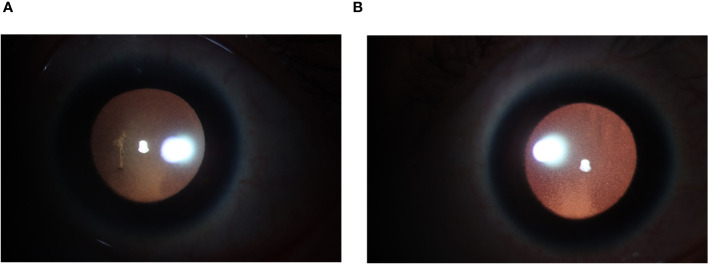
**(A)** Anterior segment photograph (L): Pupil dilated, diameter approximately 6mm. KP(+++), with anterior chamber exudation. **(B)** Anterior segment photograph (R): Pupil dilated, diameter approximately 6mm, KP (+++).

On the second day after the third cycle of BV and the first use of Tirelizumab, the patient experienced significant vision loss, conjunctival congestion, blurred vision, and photophobia. Visual acuity was severely impaired, and bilateral uveitis was diagnosed (**Figure 3**). The cerebrospinal fluid analysis showed regular reminders of small lymphocytes, with no prominent abnormal lymphoid cells observed via flow cytometry, thereby ruling out intracranial metastasis of lymphoma for the time being. Tobashi eye drops, Diclofenac Sodium Eye Drops, and atropine eye ointment were topically applied to manage the uveitis. After the discontinuation of BV, the symptoms gradually improved over a period of four weeks with the use of topical corticosteroids and mydriatic agents, leading to significant reduction in inflammation and discomfort. Follow-up examinations at two and four weeks post-discontinuation showed a marked decrease in anterior chamber cells and flare. Considering the potential for uveitis as a drug-related adverse effect, the patient’s treatment was then switched to Tislelizumab combined with the Gemox regimen (gemcitabine and oxaliplatin). After two and four cycles, PET/CT confirmed CR. However, seven months later, the patient noticed swollen lymph nodes in the left neck. PET/CT showed enlarged left supraclavicular lymph nodes with increased glucose metabolism (SUV max=6.48), indicating tumor recurrence. A cervical lymph node biopsy confirmed relapse of classical Hodgkin’s lymphoma, nodular sclerosis subtype.

The patient then received four cycles of Sindellizumab combined with the ICE regimen (ifosfamide, carboplatin, etoposide), achieving CR. On May 10, 2024, the patient underwent autologous peripheral blood stem cell transplantation and is currently scheduled for outpatient follow-up.

## Discussion

In this report, we present another important and rare case of bilateral anterior and intermediate acute uveitis in an adolescent with refractory Hodgkin’s lymphoma undergoing BV therapy. Possible causes for the uveitis included BV reuse, PD-1 inhibitors, or intraocular metastasis. From a temporal perspective, the patient experienced recurrent ocular symptoms following BV treatment, which were alleviated with local therapy. Since the removal of BV from the treatment regimen, the patient has not experienced any further episodes of uveitis. Additionally, routine examinations, including cerebrospinal fluid analysis, biochemistry, exfoliative cytology, and flow cytometry, did not indicate lymphoma metastasis. A retinal biopsy was not performed due to its invasive nature. Hou-Ting Kuo et al. reported that ICI therapies can lead to ocular immune-related adverse events (IRAEs), such as uveitis, due to the reprogramming of immune checkpoints, which can alter immune responses and affect various tissues, including ocular structures (14). During ongoing PD-1 therapy after discontinuation of BV, the patient did not experience any further episodes of uveitis. This suggests that BV was the main factor responsible for the uveitis.

In previous 7 cases of BV-induced ocular adverse events (**Table 1**) (4–8), similar to previous case reports, symptoms in our patient appeared more than two weeks after the first administration of BV, with photophobia often being the initial sign. Treatment involved discontinuing BV and using topical or systemic steroids. Vogt-Koyanagi-Harada syndrome (VKH syndrome) is a rare autoimmune disorder with bilateral granulomatous panuveitis and additional neurological, auditory, and skin symptoms (15). Therssen S et al. reported VKH-like uveitis that recurred upon reapplication of BV (8). Phylactic antiviral therapy should be considered for at-risk patients, as demonstrated by Tudesq JJ et al. who reported four cases of retinitis during BV treatment, with three of these cases linked to cytomegalovirus (CMV) infection (6). These cases underscore the potential link between BV and ocular side effects and the need for careful monitoring and management.

**Table 1:** Characteristics about the 8 ocular adverse events associated with Brentuximab vedotin.

Age	Gender	Infectious Etiology	Pathological type	Ocular adverse type	Onset Time After BV	Clinical Symptoms	Treatment
71y	F	Unknown	Relapsed cHL	VKH like granulomatous pan uveitis	3 weeks after the first use	Visual loss and floaters in both eyes, slight photophobia	Oral and topical steroids vitrectomy
33y	M	EBV+	Relapsed cHL(nodular sclerosis)	bilateral panuveitis	15 days after the first use	Pain,photophobia, and bilateral foreign body sensation	Topical steroids and pupil dilating drops Diagnostic pars plana vitrectomy of the right eye
19y	F	Unknown	HL	bilateral severe anterior and intermediate uveitis	2 weeks After the 7^th^ course	Ocular pain, photophobia, redness and blurred vision in both eyes	Topical steroids, cycloplegic agents and pupil dilating drops. Oral and intravenous prednisone
36y	F	Unknown	cHL	Purtscher-like retinopathy(occlusive microvasculo-Pathy)	3 weeks after the first use	Bilateral visual loss	IV methylprednisolone Intravitreal aflibecept injection
83y	M	CMV IgG+	HL(mixed-cellularity type)	left eye retinitis	13 days after the 3^th^ BV	Left vision loss	Ganciclovir treatment (10 mg/kg/d) for 2 weeks. valganciclovir (1800 mg/d for 7 days and then 900 mg/d)
43y	M	CMV IgG+ CMV DNA+	HL (mixed-cellularity type)	left necrotic and hemorrhagic chorio retinitis	20 days after the 4^th^ course	Left vision loss	Intravitreal and intravenous ganciclovir (10 mg/kg/d) Foscarnet (120 mg/kg/d) for 3 weeks Valganciclovir (900 mg/d)
44y	M	CMV IgG+ CMV DNA+	Stage IV peripheral T-cell lymphoma	bilateral retinitis	2 weeks after the 2^th^ course	Bilateral vision loss	Valganciclovir (1800 mg/d) Intravenous and twice-a-week intravitreal ganciclovir
16y	M	EBV-	Relapsed cHL(nodular sclerosis)	bilateral severe anterior and intermediate uveitis	4 weeks after the first use	Photophobia and conjunctival redness	topical corticosteroid drops Pupil dilating drops

y, Years; F, Female; M, Male; EBV+, Epstein-Barr Virus positive; EBV-, Epstein-Barr Virus negative; CMV IgG+, Cytomegalovirus Immunoglobulin G positive; CMV DNA+, Cytomegalovirus DNA positive; cHL, Classical Hodgkin Lymphoma; HL, Hodgkin Lymphoma; VKH, Vogt-Koyanagi-Harada; BV, Brentuximab Vedotin; IV, Intravenous; IPS, International Prognostic Score.

The mechanisms behind BV-induced uveitis may involve two main factors. One is the disruption of the balance between regulatory and inflammatory T cells due to BV’s cytotoxic effect on CD30+ T cells. Therssen et al. proposed that BV’s direct cytotoxic effect on CD30+ T cells in the uveal tissue could disrupt the balance between regulatory and inflammatory T cells, leading to increased inflammation (8). Additionally, the MMAE component disrupts microtubule dynamics in ocular cells, leading to inflammation through immune responses. Similar to BV, other MMAE-based ADCs such as Enfortumab Vedotin and Tisotumab Vedotin have also been reported to cause uveitis, suggesting that the MMAE component might be responsible for ocular adverse effects. Enfortumab Vedotin (Padcev) targets Nectin-4 and is linked to blurry vision, dry eye, and the first case of bilateral anterior subcapsular cataract (16). Tisotumab Vedotin (Tivdak) targets Tissue Factor and has been associated with conjunctivitis and dry eye in Japanese patients (17). BV cause uveitis and retinitis, with a total of eight reported cases of ocular adverse effects, including this one. These findings indicate that the ocular adverse events are likely related to the MMAE payload rather than the specific target antigen. These potential mechanisms are based on limited reports, and more research is needed to fully understand and develop preventive strategies.

## Conclusion

In conclusion, this case highlights the occurrence of acute uveitis as a rare adverse reaction associated with BV in a patient with refractory Hodgkin lymphoma. Previous reports have summarized the clinical features and potential mechanisms of BV-induced ocular adverse events. According to the ASCO guidelines (18), it is essential for clinicians to remain vigilant in detecting and managing these reactions during BV therapy. Timely evaluation, appropriate discontinuation of the drug, and treatment adjustments are crucial for optimizing patient management and prognosis. Continued case reporting is necessary to further elucidate the mechanisms of BV-induced uveitis and improve clinical management practices.

## Data availability statement

The original contributions presented in the study are included in the article/supplementary material. Further inquiries can be directed to the corresponding author.

## Ethics statement

Written informed consent was obtained from the individual(s), and minor(s)’ legal guardian/next of kin, for the publication of any potentially identifiable images or data included in this article. Written informed consent was obtained from the participant/patient(s) for the publication of this case report.

## Author contributions

ML: Writing – original draft. KR: Writing – review & editing. PA: Writing – review & editing. LZ: Funding acquisition, Investigation, Supervision, Validation, Visualization, Writing – review & editing.
